# Mass Spectrometry-Based Proteomic Profiling of Thrombotic Material Obtained by Endovascular Thrombectomy in Patients with Ischemic Stroke

**DOI:** 10.3390/ijms19020498

**Published:** 2018-02-07

**Authors:** Roberto Muñoz, Enrique Santamaría, Idoya Rubio, Karina Ausín, Aiora Ostolaza, Alberto Labarga, Miren Roldán, Beatriz Zandio, Sergio Mayor, Rebeca Bermejo, Mónica Mendigaña, María Herrera, Nuria Aymerich, Jorge Olier, Jaime Gállego, Maite Mendioroz, Joaquín Fernández-Irigoyen

**Affiliations:** 1 Department of Neurology, Complejo Hospitalario de Navarra, Pamplona 31008, Spain; roberto.munoz.arrondo@cfnavarra.es (R.M.); idorubio@hotmail.com (I.R.); aioraostolaza@gmail.com (A.O.); bzanamo@hotmail.com (B.Z.); sergiomirneurologia@hotmail.com (S.M.); isasima@hotmail.com (M.H.); aymerichnuria8@gmail.com (N.A.); jgallegocullere@gmail.com (J.G.); 2 Clinical Neuroproteomics Laboratory, Navarrabiomed, Departamento de Salud, Universidad Pública de Navarra, IDISNA, Navarra Institute for Health Research, Pamplona 31008, Spain; esantamma@navarra.es; 3 Proteored-ISCIII, Proteomics Unit, Navarrabiomed, Departamento de Salud, Universidad Pública de Navarra, IDISNA, Navarra Institute for Health Research, Pamplona 31008, Spain; karina.ausin.perez@navarra.es; 4 Bioinformatics Laboratory, Navarrabiomed—Departamento de Salud, Universidad Pública de Navarra, IDISNA, Navarra Institute for Health Research, Pamplona 31008, Spain; alberto.labarga.gutierrez@navarra.es; 5 Neuroepigenetics Laboratory, Navarrabiomed—Departamento de Salud, Universidad Pública de Navarra, IDISNA, Navarra Institute for Health Research, Pamplona 31008, Spain; miren.roldan.arrastia@navarra.es; 6 Department of Interventional Neuroradiology, Complejo Hospitalario de Navarra, Pamplona 31008, Spain; rebeca.bermejo.garces@navarra.es (R.B.); monica.mendigana.ramos@navarra.es (M.M.); jorge.olier.arenas@cfnavarra.es (J.O.)

**Keywords:** ischemic stroke, proteomics, biomarkers, endovascular therapy, thrombus

## Abstract

Thrombotic material retrieved from acute ischemic stroke (AIS) patients represents a valuable source of biological information. In this study, we have developed a clinical proteomics workflow to characterize the protein cargo of thrombi derived from AIS patients. To analyze the thrombus proteome in a large-scale format, we developed a workflow that combines the isolation of thrombus by endovascular thrombectomy and peptide chromatographic fractionation coupled to mass-spectrometry. Using this workflow, we have characterized a specific proteomic expression profile derived from four AIS patients included in this study. Around 1600 protein species were unambiguously identified in the analyzed material. Functional bioinformatics analyses were performed, emphasizing a clustering of proteins with immunological functions as well as cardiopathy-related proteins with blood-cell dependent functions and peripheral vascular processes. In addition, we established a reference proteomic fingerprint of 341 proteins commonly detected in all patients. Protein interactome network of this subproteome revealed protein clusters involved in the interaction of fibronectin with 14-3-3 proteins, TGFβ signaling, and TCP complex network. Taken together, our data contributes to the repertoire of the human thrombus proteome, serving as a reference library to increase our knowledge about the molecular basis of thrombus derived from AIS patients, paving the way toward the establishment of a quantitative approach necessary to detect and characterize potential novel biomarkers in the stroke field.

## 1. Introduction

Stroke represents one of the top causes of mortality and adult disability worldwide [1]. Currently, secondary preventive therapy to avoid stroke recurrence is based on the etiological classification of acute ischemic stroke (AIS) following the Trial of Org 10172 in Acute Stroke Treatment (TOAST) classification [2]. Regardless of the significant diagnostic effort in the clinical setting, up to 30% of stroke patients are discharged with an undetermined or unknown etiology diagnosis [3].

Lately, blood-based biomarkers have arisen as promising tools to help in the diagnosis of stroke etiology. However, these biomarkers still lack sufficient sensitivity and specificity to be implemented in the clinical setting [4]. Hopefully, the integration of new “omics” techniques will provide a comprehensive approach to study the thrombotic processes. That will be most helpful to identify novel blood biomarkers with better predictive values in the stroke field [5].

In 2015, five randomized controlled trials demonstrated that endovascular thrombectomy was effective in patients with proximal occlusions in carotid and circle of Willis circulatory system [6,7,8,9,10]. In addition to improving stroke outcome, this novel approach makes it possible to study in vivo thrombotic material. Intra-arterial therapy, when performed with newer generation devices (mainly stent retrievers), ensures that most of the thrombotic material is retrieved, as post-recanalization digital substraction angiography shows [11]. Thus, this procedure represents an opportunity to study the structure and composition of the occlusion-causing thrombi in stroke patients.

In this scenario, our study aimed to investigate the thrombotic material retrieved from AIS patients by using a clinical proteomics approach. This thrombotic material is a valuable source to identify candidate biomarkers for stroke diagnosis, as it mirrors the actual environment in which the thrombus is organized, for example left atrial appendage in most cardioembolic strokes. Interestingly, a comprehensive consensus statement has been recently published in order to provide a rational approach for studying this valuable material [12]. Here, we performed a proteomic profiling in fresh thrombus samples obtained from AIS patients who received intra-arterial therapy with thrombectomy.

## 2. Results

### 2.1. Proteme Exploration of Human Thrombus by Mass Spectrometry

**Figure 1:**
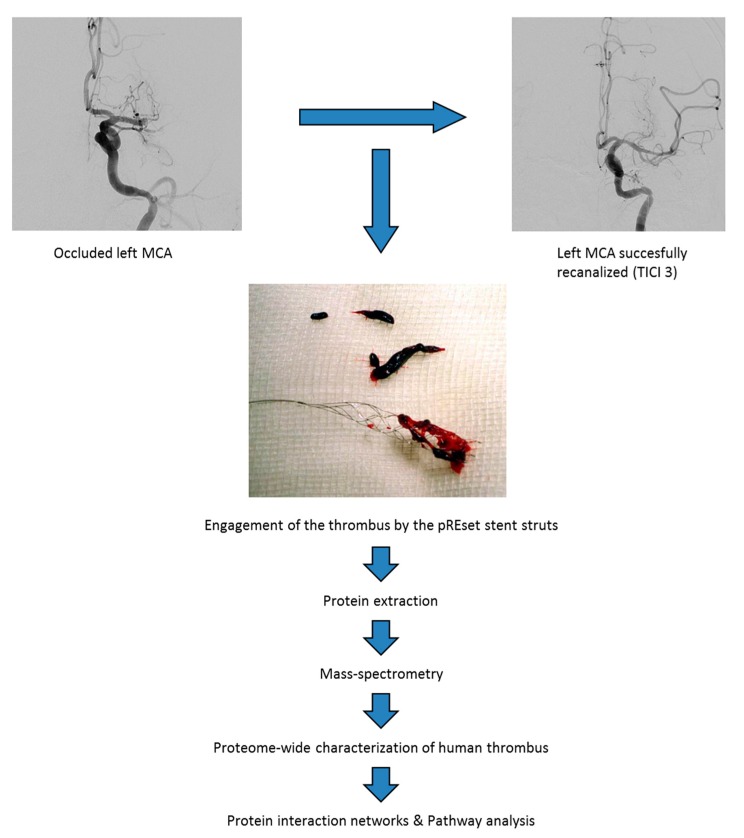
Clinical proteomics workflow applied to characterize the protein composition of human thrombus.

We have used thrombi from four AIS patients (Table 1) with the aim of obtaining a deep insight into the protein cargo and protein function of the thrombotic material isolated by endovascular thrombectomy. To this purpose, we used an integrated experimental workflow combining peptide fractionation coupled to mass spectrometry (Figure 1). The number of characterized proteins ranged from 549 to 1264 proteins, identifying around 1600 different protein species in thrombotic material. Complete lists of identifications are presented in Table S1.

### 2.2. Functional Mapping of Human Thrombotic Proteome Isolated by Endovascular Thrombectomy

**Figure 2:**
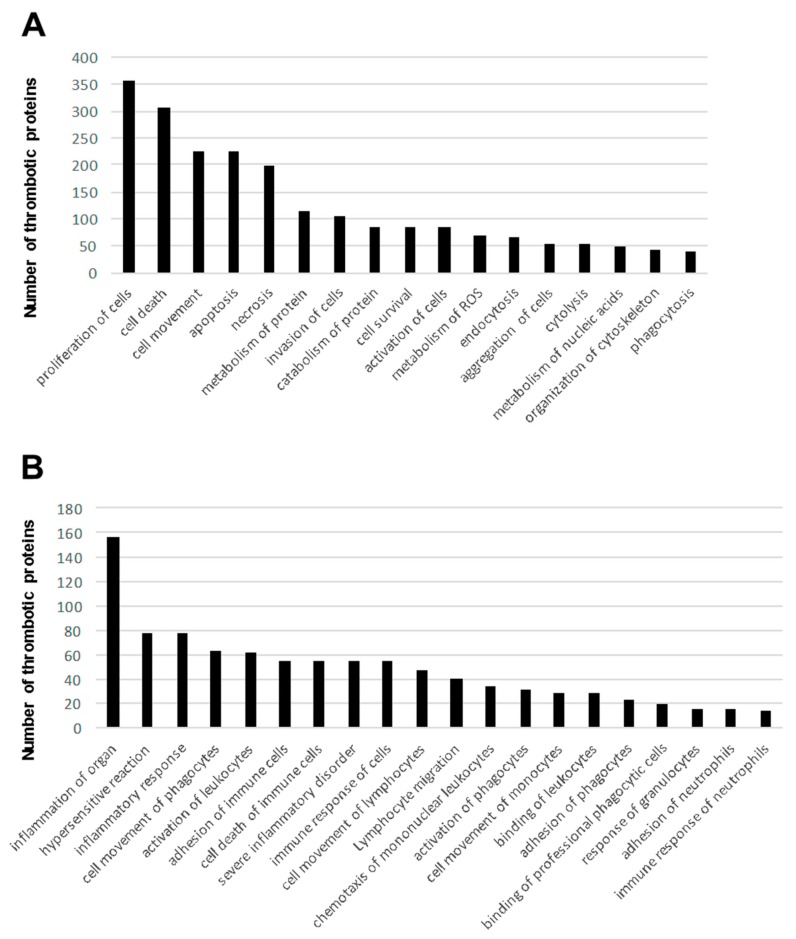
Generic (**A**) and immunological (**B**) functions significantly represented according to the thrombotic proteome characterized in this study (disease and functions from the global 1600 protein list).

**Figure 3:**
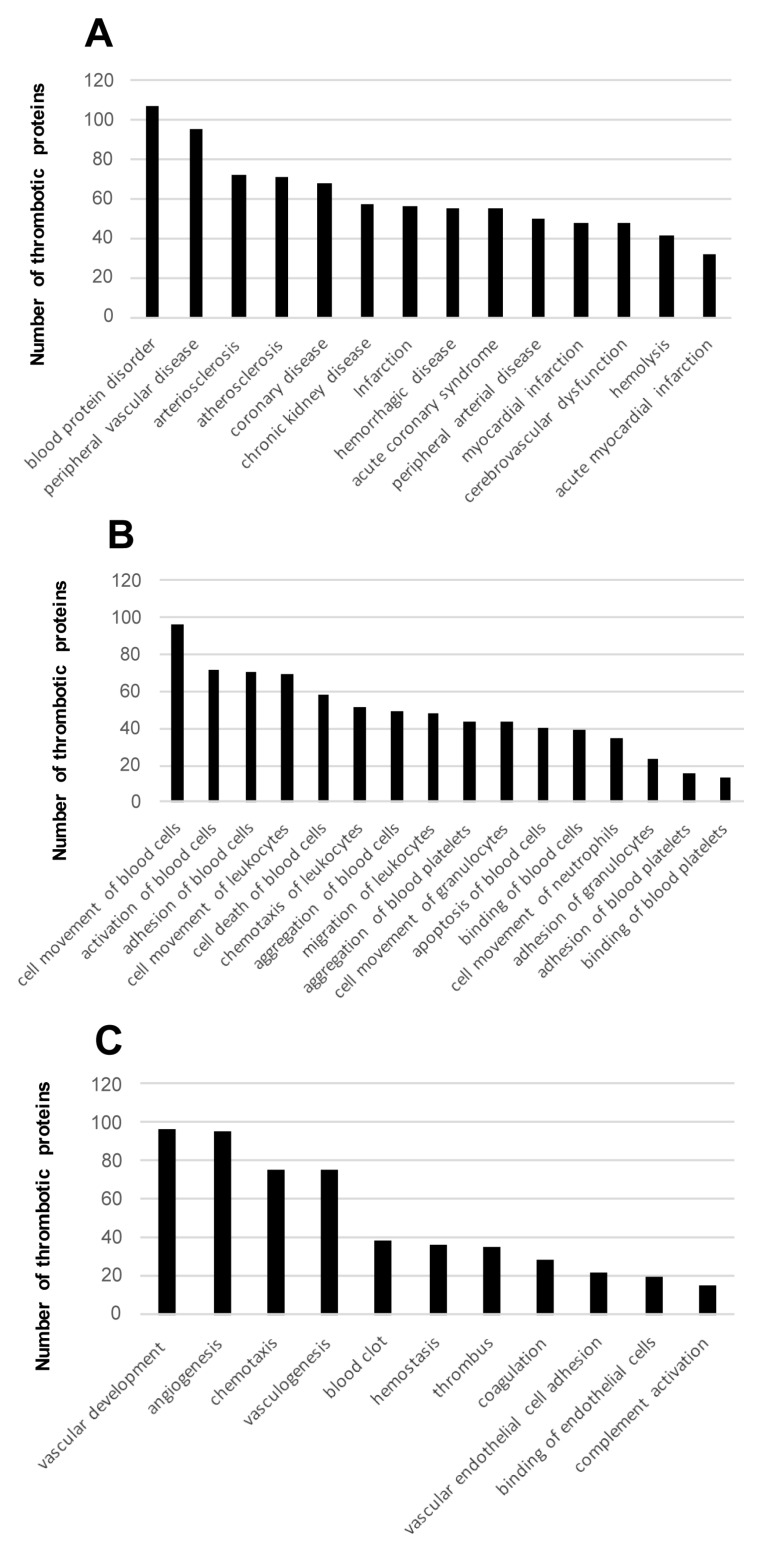
Cardiopathy-related terms (**A**), blood-cell dependent functions (**B**), and peripheral vascular processes (**C**) significantly represented according to the thrombotic proteome characterized in this study (disease and functions from the global 1600 protein list).

To interpret the proteomic fingerprints from a functional point of view, global thrombotic proteome dataset was functionally categorized. To this end, a signaling pathway characterization from all thrombotic proteins was performed with the Ingenuity Pathway Analysis (IPA) software tool (Qiagen, Redwood City, CA, USA). Some of the most significantly enriched pathways included remodeling of epithelial adherens junctions, protein ubiquitination, mitochondrial dysfunction, and acute phase response signaling, among others (Table 2 and Table S2). To deeply characterize the biological pathways represented in the thrombotic protein cargo, subsequent analyses were performed to extract the proteome distribution across molecular functions and biological processes. As shown in Figure 2A, well-known biological process and molecular disturbances involved in the development of ischemic stroke such as proliferation and cell death as well as activation and aggregation of cells, and metabolism of ROS (Table S3) were significantly over-represented. Interestingly, part of the identified proteome shows considerable immunological and cardiopathy-related functions, blood cell–dependent functions, and perivascular processes (Figure 2B and Figure 3 and Table S3). All these functions are also closely implicated in ischemic stroke pathophysiology and its downstream deleterious effects.

### 2.3. Protein Interaction Networks for Common Proteins Present in the Thrombus

**Figure 4:**
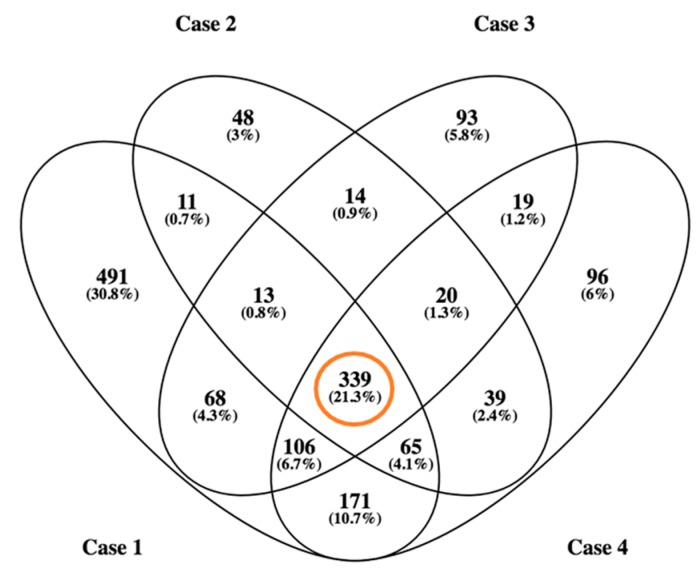
Venn diagram of identified proteins in thrombotic material obtained from the four subjects. In total, around 1600 protein species were identified.

From our global dataset, 339 proteins have been identified in all patients, comprising a “proteome reference map” (Figure 4 and Table S4). In addition, 78% of the common protein dataset (265 out of 339 protein species) has been previously detected in plasma and/or serum according to Plasma Proteome Database. Our data complements previous proteomic characterization of thrombus or its constituent platelet cells (around 6700 proteins) using similar technological approaches [13,14,15].

Interestingly, four proteins have not been previously reported associated to platelets or thrombotic material. In particular, protein-glutamine gamma-glutamyltransferase 2, Actin α cardiac muscle 1, macrophage-capping protein, and putative elongation factor 1-α-like 3 has been unambiguously identified in our sample set. Protein-glutamine gamma-glutamyltransferase 2 is an enzyme involved in the conjugation of polyamines to proteins. Actin α cardiac muscle 1 participates in cell motility. Macrophage-capping protein is a calcium-sensitive protein that reversibly blocks the pointed ends of actin filaments. The canonical function of putative elongation factor 1-α-like 3 is to bind aminoacyl-tRNA to the A-site of ribosomes in the process of protein biosynthesis. Most importantly, no relationship between any of those proteins and stroke has been previously described.

**Figure 5:**
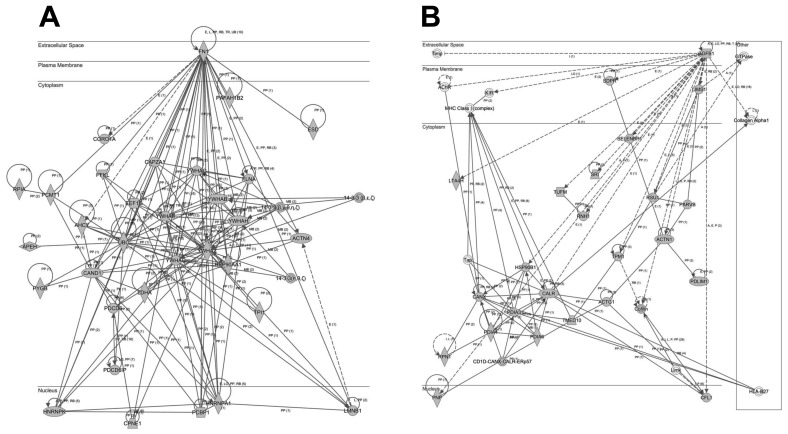
Representation of the relationships between thrombotic proteins characterized in this study: fibronectin 1 and 14-3-3 family proteins (**A**) and TGFβ signaling (**B**). Continuous lines represent direct interactions, whereas discontinuous lines correspond to indirect interactions. The complete legend may be found at http://ingenuity.force.com/ipa/articles/Feature_Description/Legend.

**Figure 6:**
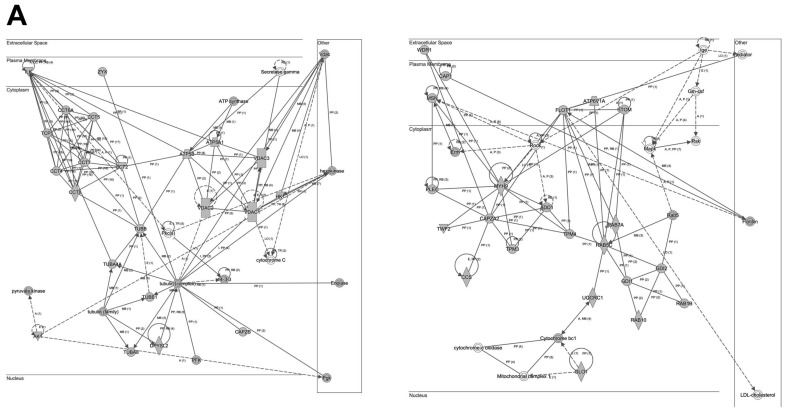

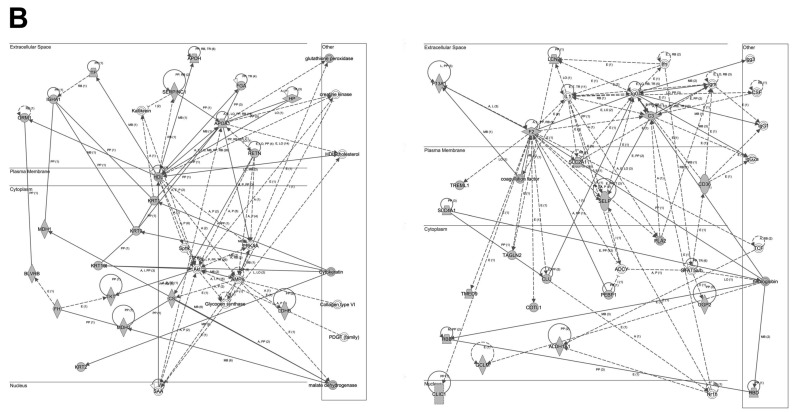
Representation of the relationships between thrombotic proteins characterized in this study: TCP complex (**A**), and lipid metabolism (**B**). Continuous lines represent direct interactions, whereas discontinuous lines correspond to indirect interactions. Continuous and discontinuous lines represent direct and indirect interactions respectively. The complete legend may be found at http://ingenuity.force.com/ipa/articles/Feature_Description/Legend.

To analyze the cooperative effects among the 341 thrombotic proteins, we constructed protein-scale interaction networks. The network-based approach allowed us to: (i) identify the molecular context of the highly connected nodes, (ii) map interactions between thrombotic proteins and functional modules, (iii) detect potential causal modulators of thrombotic protein modules that may be considered as proteins involved in thrombus formation (Figure 5 and Figure 6). As shown in Figure 5, the main axis in the network (A) corresponds to the functional relationship between fibronectin 1 and 14-3-3 family proteins. Additionally, TGFβ signaling is also over-represented in the second network (B). Other functional interactomes also suggest the involvement of TCP complex in the thrombus biology (Figure 6A).

## 3. Discussion

Three years ago, a human proteome map was made available that comprised proteins and polypeptides resulting from the activity of 17,294 genes [16]. Based on these discoveries, the rapid development of proteomic techniques has boosted novel approaches to detect and identify specific proteomes with potential impact in clinical research [17]. Thus, the use of proteomic procedures is on the rise, as much for diagnosis as for gaining insight into pathological mechanisms. In the field of stroke, there is also an increasing interest in benefiting from proteomics, on the one hand, to identify biomarkers potentially useful for diagnosis, and on the other hand, to unveil new molecular pathways involved in disease progression and thus identify new therapeutic targets.

In this scenario, and using a brain proteomics approach in stroke patients, Cuadrado et al. compared protein expression in different areas of brain tissue (core, penumbra and non-ischemic areas) identifying several proteins with differential expression that add knowledge of the ischemic cascade in order to identify candidate biomarkers of diagnostic, therapeutic or prognostic value [18].

Furthermore, several circulating proteins have been identified as potentially useful biomarkers [19,20,21]. Recently, Lind et al. developed a targeted proteomics chip including three circulating proteins (NT-pro-BNP, adrenomedullin, and eosinophil cationic protein) that could predict incident ischaemic stroke in two independent Swedish cohorts of adults aged over 70 years, independently of established cardiovascular risk factors and prevalent atrial fibrillation. This study confirms large-scale proteomics studies as a novel and exciting tool which is particularly significant in the search for new biomarkers in cerebrovascular disease [22]. However, those proteomic approaches have been performed on brain or peripheral blood samples, which may be affected by a number of regional or systemic processes reducing the specificity of the discovered biomarkers.

To the best of our knowledge, thorough proteomic analyses of thrombi responsible for AIS symptoms have not been reported so far. Studies usually focus on histological approaches to the thrombus cellular component, as well as on applying immunohistochemical procedures to assess the expression level of proteins previously known, or suspected, to be involved in thrombogenesis [23,24,25,26,27,28,29]. Unfortunately, those studies show contradictory findings, and some relevant information such as associations between clot histology and stroke etiology are lack using Haemoatoxilin/Eosin (H&E) staining [30]. However, immunostaining techniques can provide more precise information than H&E staining regarding the origin of thrombus as they allow the study of distinct cells such as platelets, T-cells or neutrophils [14]. As an example, a study revealed that atherothrombotic strokes showed higher CD3þ T-cell counts in intracranial thrombi than all other causes of stroke [31]. Still, they show limitations to fully uncover the molecular basis of ischemic stroke.

As shown in the Figure S1, high heterogeneity in cellular and fibrin content is clearly observed inter- and intra-group in our samples. On the other hand, no vascular structures are shown into the thrombus material, so we may infer that no mature thrombus has been included in our study. As previously mentioned [12], heterogeneity of methods of quantitative analysis between different studies and mainly heterogeneity of thrombus composition (composition may differ greatly in a few millimeters between two contiguous sections of the thrombus) makes correlation between thrombus components (RBCs, fibrin, platelets, white blood cells) and stroke etiology very difficult to establish. However, our proteomic workflow has defined a common proteome subset of 339 proteins independently of the thrombus origin, indicating that future quantitative proteomics approaches might complement histology analysis at the time of characterizing the thrombi origin.

It must be remarked that Alonso-Orgaz et al. have recently reported the proteome profiling of coronary thrombus [13]. By following an approach in line with ours, they were able to identify a group of proteins, i.e., myosin-9, ras-related protein Rap-1b, fermitin homolog 3, β parvin, and thrombospondin-1, whose functional features suggested that platelet focal adhesion mechanisms were involved in thrombogenesis. Those findings afford us better knowledge of pathologic pathways in thrombus formation in coronary arteries.

In our study, we show, for the first time, a protocol to characterize the proteomic profiling of thrombi in AIS patients. Some of the most significantly enriched pathways found in our work included remodeling of epithelial adherens junctions, protein ubiquitination, mitochondrial dysfunction, and acute phase response signaling, among others (Table 2 and Table S2). A number of these pathways have been previously related to stroke such as protein ubiquinitation or mitochondrial dysfunction. E3 ubiquitin ligases play an important role in post-translational modifications after brain ischemic insult, being related to processes of neuronal survival and injury [32]. Moreover, neuronal excitotoxicity is known to result in calcium overload and mitochondrial dysfunction in stroke patients [33]. Finally, several molecules involved in acute phase response signaling pathway, including IL-6 or TNF-α, have been consistently related to acute ischemic stroke [34,35,36,37,38,39] and stroke recurrence [40].

Notably, activation and aggregation of cells, particularly platelets, is of utmost importance in ischemic stroke, as thrombus formation is dependent on these biological processes. In addition, stroke involves biological mechanisms that are ignited by activation of glutamate receptors that result in high levels of intracellular Ca^2+^ and formation of reactive oxygen species (ROS) [41]. Interestingly, part of the identified proteome shows relevant immunological and cardiopathy-related functions, blood cell dependent functions, as well as perivascular processes (Figure 2B and Figure 3). All these functions are also involved in ischemic stroke pathophysiology and its downstream deleterious effects.

On the other hand, our interactome characterization showed several protein networks that may be involved in the thrombus formation (Figure 5 and Figure 6), such as the functional relationship between fibronectin 1 and 14-3-3 family proteins. Additionally, TGFβ signaling is also over-represented in the second network (B). Other functional interactomes also suggest the involvement of TCP complex in the thrombus biology (Figure 6A). Notably, fibronectin was one of the first biomarkers found to predict hemorrhagic transformation of ischemic stroke [42,43] and malignant cerebral infarction [44]. Remarkably, 14-3-3 protein is involved in the platelet surface receptor, the glycoprotein (GP) Ib-IX-V complex-dependent signaling, that initiates thrombus formation [45] and 14-3-3 isoforms are known to be differentially induced to enter into the nuclei of neurons after ischemia-reperfussion in rat models [46]. In addition, TGFβ signaling is a well-known character implicated in neuroinflammation [47] and angiogenesis [48] after ischemic stroke.

Finally, it is also compelling that new data are revealed by using this clinical proteomics approach, such as the connection of ischemic stroke with remodeling of epithelial adherens junctions pathway. Although the involvement of endothelial cell junctions in stroke is well described [49], the role of epithelial cell junctions is not clear and merits further research.

### Study Limitations

We show results obtained from a limited cohort of patients, so our conclusions should be regarded cautiously as far as the generalization of results is concerned. We included two patients with confirmed cardioembolic stroke and two patients with carotid atherothrombotic occlusion and tandem medial cerebral artery occlusion in order to cover the two most important causes of AIS.

We are aware that in situ propagation of the thrombus could modify the original thrombus or what we call “head” of the thrombus. However, the short time between stroke onset and recanalization would minimize in situ thrombus formation. Moreover, the thrombi were almost entirely extracted with the stent-retrieved, as TICI grades were compatible with complete recanalization, so we assume that this sample is representative of the actual composition of the thrombus. It might be also possible that the retrieving procedure could minimally change its protein content, modifying the results in our study, since manipulation with catheters might have induced some thrombi; however, thromboembolic complications are reported in only 1% to 2% of cerebral angiographies [50].

On the other hand, we consider that drug therapies used before the mechanical thrombectomy, i.e., pre-treatment with ASA, and especially with recombinant tissue plasminogen activator (rtPA), could also modify the thrombus proteome. However, patients had been previously treated with rtPA (Table 1) as current guidelines recommendations [51], and this fact precluded us to investigate the possible effect of thrombolytic agent in protein expression. Moreover, limited half-life of rtPA and the short time between rtPA administration and thrombus extraction would prevent significant changes in protein expression in our samples.

## 4. Materials and Methods

### 4.1. Ethics Considerations

A written consent form was obtained for research purposes from all patients included in this study, according to the Spanish Law 14/2007 of Biomedical Research, and approved (31 August 2015) by the Ethics Committee of the Complejo Hospitalario de Navarra.

### 4.2. Endovascular Thrombectomy

We selected four patients who had suffered an ischemic stroke and who had been treated with endovascular procedures by following the guidelines available at present [52]. Stent-retrievers were used to extract the thrombus. Endovascular procedures were performed through the femoral artery under general anesthesia. After placing a femoral sheath, diagnostic cerebral angiography was performed to visualize the occlusion site and the collateral channels. Afterwards, a guiding catheter was placed in. Using selective angiography with a microcatheter, the occlusion clot was visualized. The microcatheter then crossed the clot and was placed distally as previously described [53]. A Trevo™ stent was advanced and fully deployed distal to the clot. The stent was kept there for about 1–5 min to fully expand into the clot. Finally, the device with the thrombus trapped was removed slowly, maintaining a balloon inflated in distal internal carotid artery as well as continuous negative suction in order to prevent distal migration of thrombotic material during retrieval.

The endovascular device was replaced as many times as necessary to remove the thrombus. The modified thrombosis in cerebral infarction (TICI) scale was used to determine the extent of recanalization [54]. Complete recanalization was defined as TICI grades 2b or 3.

### 4.3. Periprocedural Work-Up

Cardiovascular risk factors, clinical status, and National Institutes of Health Stroke Scale (NIHSS) scores were recorded by stroke expert neurologists immediately before and after endovascular procedures. Trial of Org 10172 in Acute Stroke Treatment (TOAST) classification system was used for etiological classification.

### 4.4. Processing of Thrombus Material

Each thrombus was washed with cold saline solution in situ and stored at 4 °C until it was further processed. Then, thrombi were washed with phosphate buffered saline (PBS) solution and weighed. For their analysis, thrombi were divided in two pieces, one part showing greater thickness and more compact texture, which we called the “head” of the thrombus, while the rest was named the “tail” of the thrombus. We assumed that the head was the original thrombus, with potential information about the origin, and the tail of the thrombus may mostly consist of fresh thrombus formed in situ, which would therefore be less informative. Then, we divided the head of the thrombus into five similar pieces, four of which were frozen at −80 °C until they were processed and the last one was fixed in paraffin. The proteomic analysis was performed using the frozen samples.

### 4.5. Sample Preparation for Proteomic Analysis

Thrombotic material was homogenized in lysis buffer (7 M urea, 2 M thiourea, 4% (*v/v*) CHAPS, 50 mM DTT). The homogenates were spinned down at 100,000× *g* by ultracentifugation (1 h at 15 °C). Bradford assay (Bio-rad) was used to estimate the protein concentration.

### 4.6. Protein Analysis by LC-MS/MS

Protein extracts were precipitated with methanol/choloroform, and pellets dissolved in 6 M Urea, Tris 100 mM pH 7.8. 10 ug of protein was enzymatic cleavage with trypsin as previously described [55] and the resulting peptides were separated by by reverse phase chromatography using an Eksigent nanoLC ultra 2D pump fitted with a 75 μm ID column (Eksigent 0.075 × 250). The sample load and desalting, column gradient, and mass spectrometer setup have been described previously in detail [56].

### 4.7. Peptide Identification

MS/MS data were obtained using AnalystTF 1.5.2 (Sciex, Washington, WA, USA), and spectra files were processed through Protein Pilot TM Software 5.0 (Sciex, Washington, WA, USA) using ParagonTM Algorithm 5.0 [57] and searched against the concatenated target-decoy UniProt human database. False discovery rate was performed as previously described [58], and mass spectrometry raw data and the associated results have been deposited to the ProteomeXchange Consortium (http://proteomecentral.proteomexchange.org) via the PRIDE partner repository [59] with the dataset identifiers PXD007666 (Reviewer account details: Username: reviewer70650@ebi.ac.uk; Password: d5yK9ciY).

### 4.8. Bioinformatic Analysis

Proteomic data was analyzed by using Ingenuity^®^ Pathway Analysis (IPA) tool (QIAGEN Redwood City, CA, USA). This software helps to uncover highly represented functions and pathways in omics datasets, along with revealing interactome networks. The IPA comparison analysis reports a hierarchical ranking of signaling pathways based on the calculated *p*-value. The software generates *p*-values by using Fisher’s exact test (*p* ≤ 0.05) on the comparison between each biological or molecular event and the proteins based. To perform Venn diagrams, we have used the Venny tool (http://bioinfogp.cnb.csic.es/tools/venny/index.html).

## 5. Conclusions

In the current report, we used a discovery platform combining endovascular thrombectomy, qualitative proteomics, and physical/functional interaction data to determine the molecular composition of thrombi of human origin in ischemic stroke. On the whole, our results proved that proteomic studies in a target “tissue” of ischemic stroke are feasible and deliver important data to enrich our understanding of stroke physiopathology. In particular, thrombi retrieved with new endovascular devices in AIS patients provided us with an interesting material revealing robust biological and molecular information, such as the reference proteomic fingerprint of human thrombus described above.

One of the advantages of analyzing this thrombotic material may be the identification of specific biomarkers related to the pathophysiology of thrombus formation, given that this material mirrors the environment where the thrombus has been formed. In this context, our study opens the way for the establishment of a quantitative approach necessary to detect and characterize potential novel biomarkers in the stroke field. Hence, thrombus proteomics represents a promising perspective to explore the mechanisms underlying ischemic stroke from the inside and to identify future stroke biomarkers.

## Figures and Tables

**Table 1:** Neuroimaging and clinical characteristics.

Clinical variables	Case 1	Case 2	Case 3	Case 4
Age (years)	72	50	78	70
Gender	male	male	male	male
Stroke subtype	CE ^1^	AT ^2^	AT ^2^	CE ^1^
Hypertension	+	−	+	+
Dyslipemia	+	−	+	−
Diabetes Mellitus	−	−	+	−
Atrial Fibrilation	+	−	−	+
Ipsilateral internal carotid status	non stenosing plaques	occlusion	50–69% stenosis	non stenosing plaques
Smoke	+	+	+	+
Stroke Therapy	ev Rtpa ^3^ + Thrombectomy	Rtpa ^3^ + Thrombectomy + Angioplasty/Stenting	ev Rtpa ^3^ + Thrombectomy	ev Rtpa ^3^ + Thrombectomy
Onset to recanalization time (min)	210	360	320	210
Arterial Segment	Left MCA ^4^	Left ICA + MCA ^4^	Right MCA ^4^	Left MCA ^4^ + left ACA ^5^
Stent Retriever Dispositive	pREset	pREset	pREset	pREset
Number of passes ^6^	3	1	1	2(MCA ^4^) + 2(ACA ^5^)
TICI ^7^ scale	3	3	3	3

^1^ CE: cardioembolic stroke; ^2^ AT: atherotrombotic stroke; ^3^ Rtpa: recombinant Tissue Plasminogen Activator; ^4^ MCA: medial cerebral artery; ^5^ ACA: anterior cerebral artery; ^6^ Number of passes: number of passes performed with the stent retriever dispositive until recanalization; ^7^ TICI scale: Thrombolysis in Cerebral Infarction scale.

**Table 2:** Significantly enriched pathways in our (global) thrombotic proteome dataset.

Canonical Pathways	-Log (*p*-Value)	%	Thrombotic Proteins
Remodeling of Epithelial Adherens Junctions	2.51 × 10^−1^	56.1	37
Protein Ubiquitination Pathway	2.47 × 10^−1^	28.3	72
Mitochondrial Dysfunction	2.36 × 10^−1^	33.9	56
Acute Phase Response Signaling	2.23 × 10^−1^	32.7	55
Clathrin-mediated Endocytosis Signaling	2.18 × 10^−1^	30.1	59
Integrin Signaling	2.14 × 10^−1^	28.8	61
Caveolar-mediated Endocytosis Signaling	2.02 × 10^−1^	47.9	34
Phagosome Maturation	1.97 × 10^−1^	34.3	46
LXR/RXR Activation	1.83 × 10^−1^	34.7	42
Actin Cytoskeleton Signaling	1.82 × 10^−1^	26.1	58
Fcγ Receptor-mediated Phagocytosis in Macrophages and Monocytes	1.75 × 10^−1^	38.7	36
RhoGDI Signaling	1.49 × 10^−1^	26.7	46
Leukocyte Extravasation Signaling	1.44 × 10^−1^	24.4	50
Ephrin Receptor Signaling	1.42 × 10^−1^	26.2	45
Gap Junction Signaling	1.37 × 10^−1^	26.2	43
NRF2-mediated Oxidative Stress Response	1.32 × 10^−1^	24.2	46

## Supplementary Materials

Supplementary materials can be found at http://www.mdpi.com/1422-0067/19/2/498/s1.

## References

[1] Mukherjee D., Patil C.G. (2011). Epidemiology and the global burden of Stroke. World Neurosurg..

[2] Adams H.P., Bendixen B.H., Kappelle L.J., Biller J., Love B.B., Gordon D.L., Marsh E.E. (1993). Classification of subtype of acute ischemic Stroke. Definitions for use in a multicenter clinical trial. TOAST. Trial of Org 10172 in Acute Stroke Treatment. Stroke.

[3] Ionita C.C., Xavier A.R., Kirmani J.F., Dash S., Divani A.A., Qureshi A.I. (2005). What proportion of Stroke is not explained by classic risk factors?. Prev. Cardiol..

[4] Jickling G.C., Sharp F.R. (2015). Biomarker panels in ischemic Stroke. Stroke.

[5] Liumbruno G.M., Franchini M. (2013). Proteomic analysis of venous thromboembolism: An update. Expert Rev. Proteom..

[6] Berkhemer O.A., Fransen P.S., Beumer D., van den Berg L.A., Lingsma H.F., Yoo A.J., Schonewille W.J., Vos J.A., Nederkoorn P.J., Wermer M.J. (2015). A randomized trial of intraarterial treatment for acute ischemic Stroke. N. Engl. J. Med..

[7] Campbell B.C., Mitchell P.J., Kleinig T.J., Dewey H.M., Churilov L., Yassi N., Yan B., Dowling R.J., Parsons M.W., Oxley T.J. (2015). Endovascular therapy for ischemic Stroke with perfusion-imaging selection. N. Engl. J. Med..

[8] Goyal M., Demchuk A.M., Menon B.K., Eesa M., Rempel J.L., Thornton J., Roy D., Jovin T.G., Willinsky R.A., Sapkota B.L. (2015). Randomized assessment of rapid endovascular treatment of ischemic Stroke. N. Engl. J. Med..

[9] Jovin T.G., Chamorro A., Cobo E., de Miquel M.A., Molina C.A., Rovira A., San Roman L., Serena J., Abilleira S., Ribo M. (2015). Thrombectomy within 8 hours after symptom onset in ischemic Stroke. N. Engl. J. Med..

[10] Saver J.L., Goyal M., Bonafe A., Diener H.C., Levy E.I., Pereira V.M., Albers G.W., Cognard C., Cohen D.J., Hacke W. (2015). Stent-retriever thrombectomy after intravenous t-PA vs. t-PA alone in Stroke. N. Engl. J. Med..

[11] Goyal M., Menon B.K., van Zwam W.H., Dippel D.W., Mitchell P.J., Demchuk A.M., Davalos A., Majoie C.B., van der Lugt A., de Miquel M.A. (2016). Endovascular thrombectomy after large-vessel ischaemic Stroke: A meta-analysis of individual patient data from five randomised trials. Lancet.

[12] De Meyer S.F., Andersson T., Baxter B., Bendszus M., Brouwer P., Brinjikji W., Campbell B.C., Costalat V., Davalos A., Demchuk A. (2017). Analyses of thrombi in acute ischemic Stroke: A consensus statement on current knowledge and future directions. Int. J. Stroke.

[13] Alonso-Orgaz S., Moreno-Luna R., Lopez J.A., Gil-Dones F., Padial L.R., Moreu J., de la Cuesta F., Barderas M.G. (2014). Proteomic characterization of human coronary thrombus in patients with ST-segment elevation acute myocardial infarction. J. Proteom..

[14] Burkhart J.M., Vaudel M., Gambaryan S., Radau S., Walter U., Martens L., Geiger J., Sickmann A., Zahedi R.P. (2012). The first comprehensive and quantitative analysis of human platelet protein composition allows the comparative analysis of structural and functional pathways. Blood.

[15] Lee H., Chae S., Park J., Bae J., Go E.B., Kim S.J., Kim H., Hwang D., Lee S.W., Lee S.Y. (2016). Comprehensive Proteome Profiling of Platelet Identified a Protein Profile Predictive of Responses to an Antiplatelet Agent Sarpogrelate. Mol. Cell. Proteom..

[16] Kim M.S., Pinto S.M., Getnet D., Nirujogi R.S., Manda S.S., Chaerkady R., Madugundu A.K., Kelkar D.S., Isserlin R., Jain S. (2014). A draft map of the human proteome. Nature.

[17] Aebersold R., Mann M. (2016). Mass-spectrometric exploration of proteome structure and function. Nature.

[18] Cuadrado E., Rosell A., Colome N., Hernandez-Guillamon M., Garcia-Berrocoso T., Ribo M., Alcazar A., Ortega-Aznar A., Salinas M., Canals F. (2010). The proteome of human brain after ischemic Stroke. J. Neuropathol. Exp. Neurol..

[19] Maestrini I., Ducroquet A., Moulin S., Leys D., Cordonnier C., Bordet R. (2016). Blood biomarkers in the early stage of cerebral ischemia. Rev. Neurol..

[20] Piccardi B., Giralt D., Bustamante A., Llombart V., Garcia-Berrocoso T., Inzitari D., Montaner J. (2017). Blood markers of inflammation and endothelial dysfunction in cardioembolic Stroke: Systematic review and meta-analysis. Biomarkers.

[21] Prentice R.L., Paczesny S., Aragaki A., Amon L.M., Chen L., Pitteri S.J., McIntosh M., Wang P., Buson Busald T., Hsia J. (2010). Novel proteins associated with risk for coronary heart disease or Stroke among postmenopausal women identified by in-depth plasma proteome profiling. Genome Med..

[22] Lind L., Siegbahn A., Lindahl B., Stenemo M., Sundstrom J., Arnlov J. (2015). Discovery of new risk markers for ischemic Stroke using a novel targeted proteomics chip. Stroke.

[23] Almekhlafi M.A., Hu W.Y., Hill M.D., Auer R.N. (2008). Calcification and endothelialization of thrombi in acute Stroke. Ann. Neurol..

[24] Boeckh-Behrens T., Kleine J.F., Zimmer C., Neff F., Scheipl F., Pelisek J., Schirmer L., Nguyen K., Karatas D., Poppert H. (2016). Thrombus histology suggests cardioembolic cause in cryptogenic Stroke. Stroke.

[25] Hashimoto T., Hayakawa M., Funatsu N., Yamagami H., Satow T., Takahashi J.C., Nagatsuka K., Ishibashi-Ueda H., Kira J.I., Toyoda K. (2016). Histopathologic analysis of retrieved thrombi associated with successful reperfusion after acute Stroke thrombectomy. Stroke.

[26] Kim S.K., Yoon W., Kim T.S., Kim H.S., Heo T.W., Park M.S. (2015). Histologic analysis of retrieved clots in acute ischemic Stroke: Correlation with Stroke etiology and gradient-echo MRI. Am. J. Neuroradiol..

[27] Niesten J.M., van der Schaaf I.C., van Dam L., Vink A., Vos J.A., Schonewille W.J., de Bruin P.C., Mali W.P., Velthuis B.K. (2014). Histopathologic composition of cerebral thrombi of acute Stroke patients is correlated with Stroke subtype and thrombus attenuation. PLoS ONE.

[28] Simons N., Mitchell P., Dowling R., Gonzales M., Yan B. (2015). Thrombus composition in acute ischemic Stroke: A histopathological study of thrombus extracted by endovascular retrieval. J. Neuroradiol..

[29] Sporns P.B., Hanning U., Schwindt W., Velasco A., Minnerup J., Zoubi T., Heindel W., Jeibmann A., Niederstadt T.U. (2017). Ischemic Stroke: What does the histological composition tell us about the origin of the thrombus?. Stroke.

[30] Brinjikji W., Duffy S., Burrows A., Hacke W., Liebeskind D., Majoie C., Dippel D.W.J., Siddiqui A.H., Khatri P., Baxter B. (2017). Correlation of imaging and histopathology of thrombi in acute ischemic Stroke with etiology and outcome: A systematic review. J. Neurointerv. Surg..

[31] Dargazanli C., Rigau V., Eker O., Bareiro C.R., Machi P., Gascou G., Arquizan C., Ayrignac X., Mourand I., Corlobe A. (2016). High CD3+ Cells in Intracranial Thrombi Represent a Biomarker of Atherothrombotic Stroke. PLoS ONE.

[32] Lackovic J., Howitt J., Callaway J.K., Silke J., Bartlett P., Tan S.S. (2012). Differential regulation of Nedd4 ubiquitin ligases and their adaptor protein Ndfip1 in a rat model of ischemic Stroke. Exp. Neurol..

[33] Prentice H., Modi J.P., Wu J.Y. (2015). Mechanisms of neuronal protection against excitotoxicity, endoplasmic reticulum stress, and mitochondrial dysfunction in Stroke and neurodegenerative diseases. Oxid. Med. Cell. Longev..

[34] Castellanos M., Castillo J., Garcia M.M., Leira R., Serena J., Chamorro A., Davalos A. (2002). Inflammation-mediated damage in progressing lacunar infarctions: A potential therapeutic target. Stroke.

[35] Esenwa C.C., Elkind M.S. (2016). Inflammatory risk factors, biomarkers and associated therapy in ischaemic Stroke. Nat. Rev. Neurol..

[36] Montaner J., Salat D., Garcia-Berrocoso T., Molina C.A., Chacon P., Ribo M., Alvarez-Sabin J., Rosell A. (2010). Reperfusion therapy for acute Stroke improves outcome by decreasing neuroinflammation. Transl. Stroke Res..

[37] Rallidis L.S., Vikelis M., Panagiotakos D.B., Rizos I., Zolindaki M.G., Kaliva K., Kremastinos D.T. (2006). Inflammatory markers and in-hospital mortality in acute ischaemic Stroke. Atherosclerosis.

[38] Sairanen T., Carpen O., Karjalainen-Lindsberg M.L., Paetau A., Turpeinen U., Kaste M., Lindsberg P.J. (2001). Evolution of cerebral tumor necrosis factor-α production during human ischemic Stroke. Stroke.

[39] Vila N., Castillo J., Davalos A., Chamorro A. (2000). Proinflammatory cytokines and early neurological worsening in ischemic Stroke. Stroke.

[40] Castillo J., Alvarez-Sabin J., Martinez-Vila E., Montaner J., Sobrino T., Vivancos J. (2009). Inflammation markers and prediction of post-Stroke vascular disease recurrence: The MITICO study. J. Neurol..

[41] Juurlink B.H., Sweeney M.I. (1997). Mechanisms that result in damage during and following cerebral ischemia. Neurosci. Biobehav. Rev..

[42] Castellanos M., Leira R., Serena J., Blanco M., Pedraza S., Castillo J., Davalos A. (2004). Plasma cellular-fibronectin concentration predicts hemorrhagic transformation after thrombolytic therapy in acute ischemic Stroke. Stroke.

[43] Castellanos M., Sobrino T., Millan M., Garcia M., Arenillas J., Nombela F., Brea D., Perez de la Ossa N., Serena J., Vivancos J. (2007). Serum cellular fibronectin and matrix metalloproteinase-9 as screening biomarkers for the prediction of parenchymal hematoma after thrombolytic therapy in acute ischemic Stroke: A multicenter confirmatory study. Stroke.

[44] Serena J., Blanco M., Castellanos M., Silva Y., Vivancos J., Moro M.A., Leira R., Lizasoain I., Castillo J., Davalos A. (2005). The prediction of malignant cerebral infarction by molecular brain barrier disruption markers. Stroke.

[45] Andrews R.K., Berndt M.C. (1998). Adhesion-dependent signalling and the initiation of haemostasis and thrombosis. Histol. Histopathol..

[46] Zhou X.Y., Hu D.X., Chen R.Q., Chen X.Q., Dong W.L., Yi C.L. (2017). 14-3-3 isoforms differentially regulate NFκB signaling in the brain after ischemia-reperfusion. Neurochem. Res..

[47] Cekanaviciute E., Fathali N., Doyle K.P., Williams A.M., Han J., Buckwalter M.S. (2014). Astrocytic transforming growth factor-β signaling reduces subacute neuroinflammation after Stroke in mice. Glia.

[48] Cipollone F., Fazia M., Mincione G., Iezzi A., Pini B., Cuccurullo C., Ucchino S., Spigonardo F., di Nisio M., Cuccurullo F. (2004). Increased expression of transforming growth factor-β1 as a stabilizing factor in human atherosclerotic plaques. Stroke.

[49] Dejana E., Tournier-Lasserve E., Weinstei B.M. (2009). The control of vascular integrity by endothelial cell junctions: Molecular basis and pathological implications. Dev. Cell.

[50] Kaufmann T.J., Huston J., Mandrekar J.N., Schleck C.D., Thielen K.R., Kallmes D.F. (2007). Complications of diagnostic cerebral angiography: Evaluation of 19,826 consecutive patients. Radiology.

[51] Powers W.J., Rabinstein A.A., Ackerson T., Adeoye O.M., Bambakidis N.C., Becker K., Biller J., Brown M., Demaerschalk B.M., Hoh B. (2018). 2018 Guidelines for the early management of patients with acute ischemic Stroke: A guideline for healthcare professionals from the American Heart Association/American Stroke Association. Stroke.

[52] Powers W.J., Derdeyn C.P., Biller J., Coffey C.S., Hoh B.L., Jauch E.C., Johnston K.C., Johnston S.C., Khalessi A.A., Kidwell C.S. (2015). American Heart Association/American Stroke Association focused update of the 2013 guidelines for the early management of patients with acute ischemic stroke regarding endovascular treatment: A guideline for healthcare professionals from the American Heart Association/American Stroke Association. Stroke.

[53] Park H., Hwang G.J., Jin S.C., Jung C.K., Bang J.S., Han M.K., Bae H.J., Choe G.Y., Oh C.W., Kwon O.K. (2011). A retrieval thrombectomy technique with the Solitaire stent in a large cerebral artery occlusion. Acta Neurochir..

[54] Zaidat O.O., Yoo A.J., Khatri P., Tomsick T.A., von Kummer R., Saver J.L., Marks M.P., Prabhakaran S., Kallmes D.F., Fitzsimmons B.F. (2013). Recommendations on angiographic revascularization grading standards for acute ischemic Stroke: A consensus statement. Stroke.

[55] Shevchenko A., Tomas H., Havlis J., Olsen J.V., Mann M. (2006). In-gel digestion for mass spectrometric characterization of proteins and proteomes. Nat. Protoc..

[56] Lachen-Montes M., Gonzalez-Morales A., Zelaya M.V., Perez-Valderrama E., Ausin K., Ferrer I., Fernandez-Irigoyen J., Santamaria E. (2017). Olfactory bulb neuroproteomics reveals a chronological perturbation of survival routes and a disruption of prohibitin complex during Alzheimer’s disease progression. Sci. Rep..

[57] Shilov I.V., Seymour S.L., Patel A.A., Loboda A., Tang W.H., Keating S.P., Hunter C.L., Nuwaysir L.M., Schaeffer D.A. (2007). The Paragon Algorithm, a next generation search engine that uses sequence temperature values and feature probabilities to identify peptides from tandem mass spectra. Mol. Cell. Proteom..

[58] Tang W.H., Shilov I.V., Seymour S.L. (2008). Nonlinear fitting method for determining local false discovery rates from decoy database searches. J. Proteom. Res..

[59] Vizcaino J.A., Deutsch E.W., Wang R., Csordas A., Reisinger F., Rios D., Dianes J.A., Sun Z., Farrah T., Bandeira N. (2014). ProteomeXchange provides globally coordinated proteomics data submission and dissemination. Nat. Biotechnol..

