# Supplementary figures and images for: Mass Spectrometry-Based Proteomic Profiling of Thrombotic Material Obtained by Endovascular Thrombectomy in Patients with Ischemic Stroke
Source: Int J Mol Sci. 2018 Feb 7;19(2):498. doi: 10.3390/ijms19020498 (PMC5855720; doi:10.3390/ijms19020498)

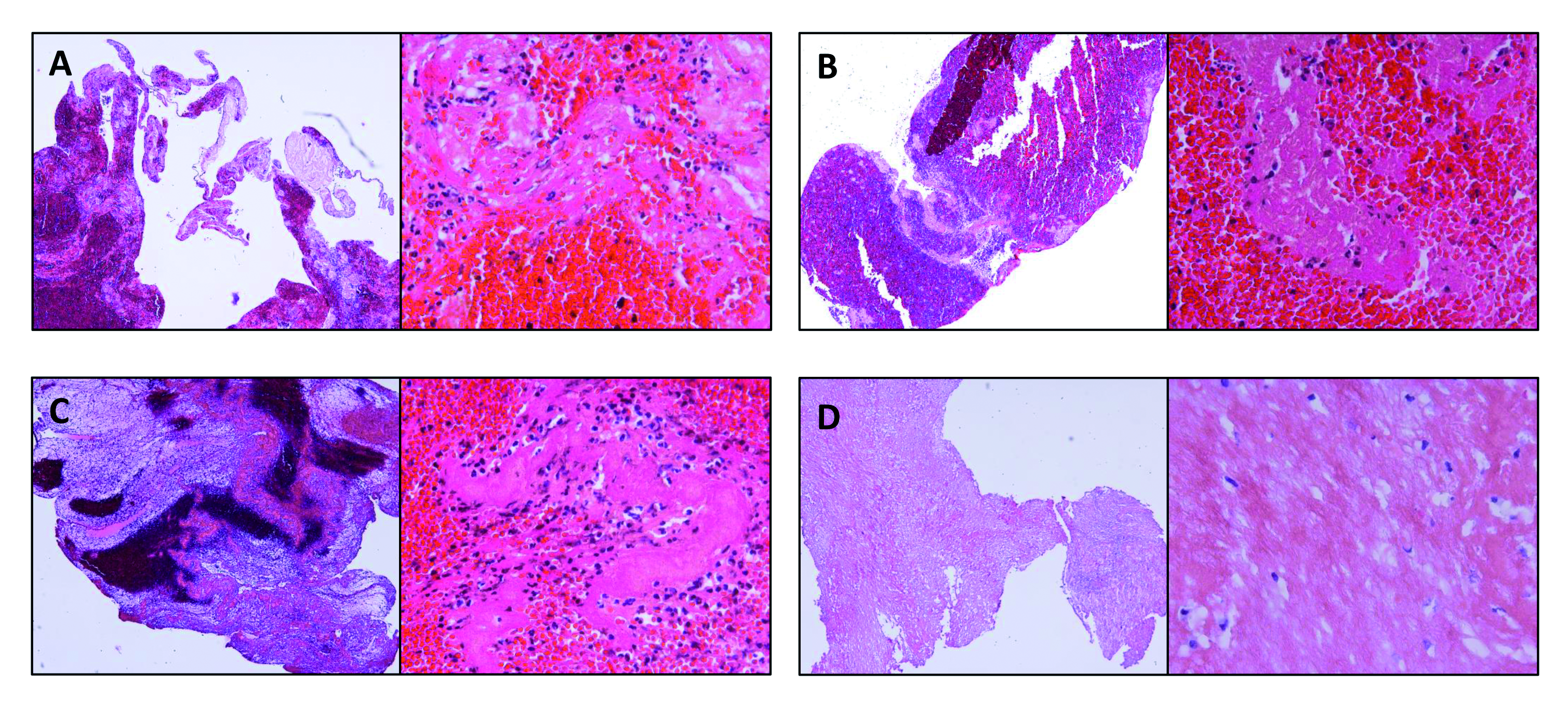

Supplement: Supplementary file 1 [file ijms-19-00498-s001.zip › ijms-260181-SI/Figure S1.tif]
